# Thermal Contact Conductance-Based Thermal Behavior Analytical Model for a Hybrid Floor at Elevated Temperatures

**DOI:** 10.3390/ma13194257

**Published:** 2020-09-24

**Authors:** Min Jae Park, Jeong Ki Min, Jaehoon Bae, Young K. Ju

**Affiliations:** 1 School of Civil, Environmental, and Architectural Engineering, Korea University, Seoul 02481, Korea; alswo8739@korea.ac.kr (M.J.P.); skycity-bjh@korea.ac.kr (J.B.); 2 Fire Protection Evaluation Technology Center, Korea Conformity Laboratories, Chungcheongbuk-do 28115, Korea; jkm927@kcl.re.kr

**Keywords:** polymeric material, hybrid floor, thermal contact conductance, thermal behavior analytical model, fire resistance performance

## Abstract

Hybrid floors infilled with polymeric materials between two steel plates were developed as a prefabricated floor system in the construction industry. However, the floor’s fire resistance performance has not been investigated. To evaluate this, fire tests suggested by the Korean Standards should be performed. As these tests are costly and time consuming, the number of variables were limited. However, many variables can be investigated in other ways such as furnace tests and finite element analysis (FEA) with less cost and time. In this study, furnace tests on heated surface areas smaller than 1 m^2^ were conducted to investigate the thermal behavior of the hybrid floor at elevated temperatures. To obtain the reliability of the proposed thermal behavior analytical (TBA) model, verifications were conducted by FEAs. Thermal contact conductance including interfacial thermal properties between two materials was adopted in the TBA model, and the values at elevated temperatures were suggested based on thermo-gravimetric analyses results and verified by FEA. Errors between the tests and TBA model indicated that the model was adequate in predicting the temperature distribution in small-scale hybrids. Furthermore, larger furnace tests and analysis results were compared to verify the TBA model’s application to different sized hybrid floors.

## 1. Introduction

**Figure 1:**
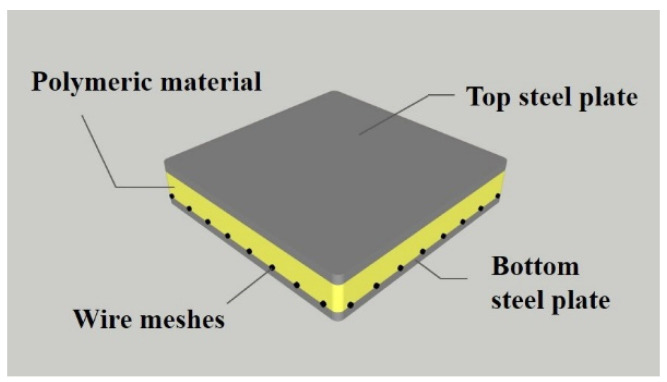
Components of the hybrid floor.

Prefabricated composite hybrid floors, which consist of polymeric materials between top and bottom steel plates, such as sandwich panels (Figure 1), were developed to apply to steel structures [1,2,3,4,5,6,7,8,9]. Because the polymeric materials in the floor have strong bond strengths with steel plates under large deformations, the hybrid floor exhibits fully composite and interaction behaviors under loading [1,2,3,10,11,12]. As bottom steel plates and bottom parts of polymeric materials lose their mechanical properties at elevated temperatures, wire meshes enhance the bending capacity of the floor. The structural performance of the floor at the ambient temperature was investigated, and the serviceability, including the floor vibration and damping ratio, was also studied [1,2,3,4,5]. The hybrid floor system with insulating materials for preventing fire were installed in two actual buildings, a church and a residential building. Floor vibration tests were conducted, and the dynamic characteristics of the buildings were investigated [5].

To be an absolute prefabricated floor system, this hybrid floor must exhibit the fire resistance performance required in the Republic of Korea. The fire resistance performances of horizontal members in buildings are evaluated by three criteria based on Korean Standards (KS) [13,14]. The stability-related deformation and rate of the deformation, insulation-related changes in temperatures, and integrity-related occurrences of flames at unheated surfaces during fire tests belong to the criteria. In the case of this floor system, fire tests were conducted on the horizontal equipment, and the lower parts of the specimen were heated by a standard-fire-heating-curve equal to the International Organization for Standardization (ISO) 834 curve [15,16]. When any criterion was unsatisfied, the time would be the fire resistance ratings of the specimen. Because there is no question of a flame appearing at the unheated surfaces of the hybrid floor, only the stability and insulation were used for evaluating the fire resistance performance of the floor. Preliminary fire tests were conducted to survey the behaviors at elevated temperatures caused by many factors such as the phase changes of polymeric materials, interfacial properties between the steel and polymeric material, and the possibility of flames in the polymeric materials [6,7,8,9]. These tests showed that many factors could predict the fire resistance performance of the hybrid floor.

**Figure 2:**
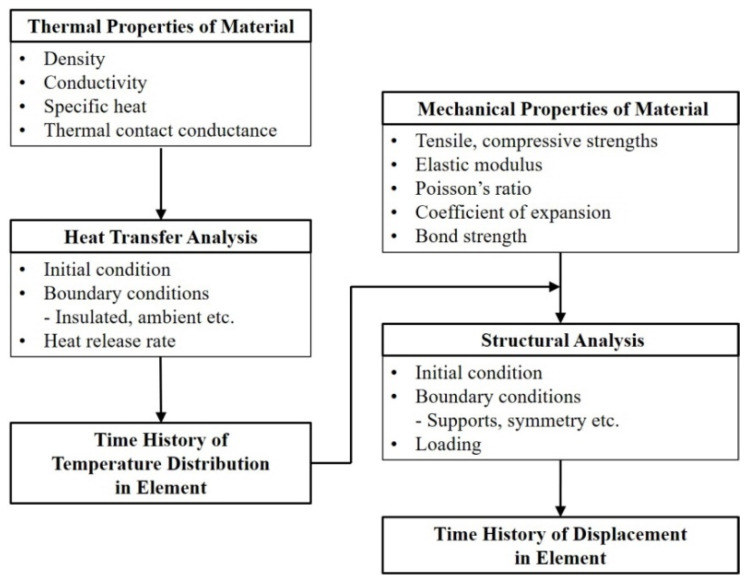
Procedure of the analytical studies for fire resistance.

The fire tests based on the KS and ISO are time intensive and costly to conduct. As the Republic of Korea has very few authorized furnaces, a long waiting time was required to conduct the tests, and few specimens could be investigated. Therefore, analytical studies or furnace tests with numerous variables were the most efficient ways to study the fire resistance performance of the hybrid floor [17,18]. The procedure for the analytical studies is shown in Figure 2. Thermal properties and the applied theories of heat transfer were quite important because the results of the heat transfer analysis determined the mechanical properties of the elements at elevated temperatures in the structural analysis. Thus, to improve the reliability of the analytical studies, accurate thermal properties including the interfacial properties of the composite members should be adopted.

**Figure 3:**
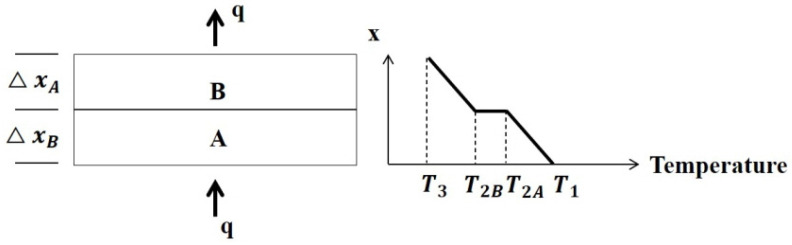
Diagram of thermal contact conductance.

Studies on thermal contact conductance (TCC), an interfacial property, have been conducted since the 1950s [19]. TCC can be measured by the temperature drops (T_2B_-T_2A_) between two materials owing to invisible interstitial gaps at the contact surfaces (A-B) as shown in Figure 3.

**Figure 4:**
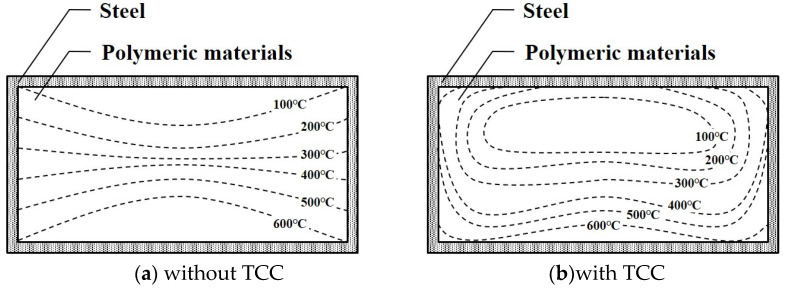
Examples of temperature distributions in hybrid floors: (**a**) without TCC (**b**) with TCC.

For building structural members, studies on TCC have been conducted by researchers since the 2000s, and only the TCC between concrete and steel were investigated [20,21,22,23,24,25,26]. From the results of these studies, many researchers applied the TCC theory and the obtained values to the analytical modeling for estimating fire resistance performance. In addition, studies about TCC between metal and polymers were performed, and these results provided the values for the analytical modeling in this study [27,28,29]. The reason TCC should be considered in evaluating the fire resistance performance of hybrid floors is because the difference in temperature distribution (Figure 4) will determine inappropriate mechanical properties and predict invalid deformations at elevated temperatures.

The aim of this study was to investigate the thermal behavior of a hybrid floor at elevated temperatures based on previous studies and propose an analytical model to predict the temperature distribution of elements that are the basis of estimations of fire resistance performance. In addition, the reliability of the proposed analytical model, a thermal contact conductance-based thermal behavior analytical (TBA) model, was enhanced by verifications using a finite element analysis (FEA) that simulated the furnace tests.

## 2. State-of-the-Art Studies

### 2.1. Hybrid Floor

The polymeric materials are a one of type of polyurethane formed by reacting a diisocyanate that contains phosphorous with a polyol mixture. The mechanical properties tests of polymeric materials were conducted based on the American Society for Testing and Materials (ASTM) and are listed in Table 1 [1,2,3]. Although the tensile, compressive strengths, and elasticity modulus of polymeric materials were much weaker than those of general steel plates, the ultimate strains of polymeric materials were much larger than those of general steel plates. Based on flexural tests of the hybrid floor, the experimental flexural strengths were 8% higher than theoretical plastic moment strengths [1,2,3]. For predicting the calibration of deformation, Kim [2] suggested that summation of theoretical bending and shear deformation should be divided by 0.85. In addition, the FEA of the floor was performed to examine the state of stress generated between the steel plates and the polymeric materials [3]. Floor vibration of the floor that had both pined supports can be predicted by Steel Construction Institute (SCI) publication [4,30]. In addition, the floor vibration tests of actual buildings were performed, and the results exhibited that the floor showed equal performance of original steel structures slabs recommended by the American Institute of Steel Construction (AISC) [31].

**Figure 5:**
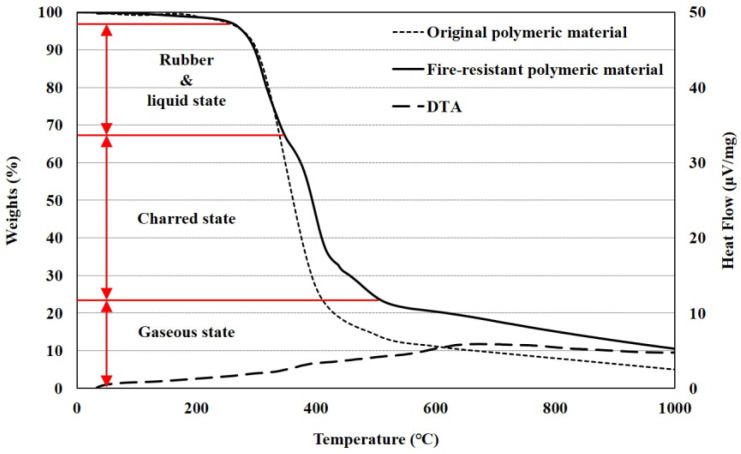
Thermal gravimetric analysis (TGA) and differential thermal analysis (DTA) results of original and fire-resistant polymeric materials.

Preliminary fire tests in accordance with KS and ISO were conducted [5,6,7]. After 26 min, the fire tests were stopped because of the spacers that were installed for improving the efficiency of the manufacturing process in the factory. From these tests results, 2~5 mm charred layer that behaved as an insulation material was revealed at the bottom part of the polymeric materials and heated through the exposed surfaces similar to the char of wood structures [32,33,34,35] and decomposition of sandwich systems [36] in fire conditions. In addition, a gap between the lower steel plates and charred layer occurred due to the air that inevitably appears in polymeric materials during the manufacturing process. These results indicate that the thermal performance of polymeric materials should be improved to be a fire-resistant structure. Finally, the fire-resistant polymeric material was developed and compared with the original materials through a thermal gravimetric analysis (TGA) as shown in Figure 5. TGA shows the residual mass for the test materials at elevated temperatures. In Figure 5, a differential thermal analysis (DTA) result that provides data on the transformations that have occurred such as phase change for fire-resistant polymeric materials were neglected because the DTA result showed that those values were very small. This meant that the latent heat of polymeric materials due to phase change can be ignored because DTA shows the latent heat of materials at elevated temperatures. The differences between the original polymeric materials and fire-resistant polymeric materials were combustibility and the sustainability of the charred state that would explain the results of the tests.

### 2.2. Thermal Contact Conductance Studies for Structural Elements

Although fire studies were conducted on various structural members, the heat transfer analysis containing basic heat transfer theory—conduction, convection, and radiation—and moisture changes did not include the interface between two materials. For this reason, the numerical studies usually exhibit more conservative results than the experimental studies [37,38]. Therefore, some researchers suggested that the TCC between two materials should be considered and that it would make the results more significant compared with those that ignore the TCC [20,21,26]. Many researchers conducted tests to determine the TCC between concrete and steel in structural members. Based on these studies, the values of the TCC range from 38.1 to 200 W/m^2^∙K [20,21,22,23,24,25,26]. However, there is no study on the TCC between polymers and steel that can be applied to a floor under fire.

### 2.3. Thermal Contact Conductance between Polymer and Metal

Because there is no study for the TCC between polymers and steel, the values of the TCC between the polymer and metal were used. Fuller and Marotta [27] proposed a TCC model of polymer/metal and verified it with experimental data. The values of the TCC obtained by the model and data were between 40 and 270 W/m^2^∙K at various contact pressures. Bahrami et al. [28] proposed the compact analytical thermal contact resistance (TCR) model that was the inverse of the TCC and verified it with 13 polymer-metal data sets containing 127 experimental data points. The values of the TCC by the compact analytical model had a range of 22–250 W/m^2^∙K at various contact pressures. Gibbins [29] suggested a TCR value of approximately 8 K/W at atmospheric pressure for a specimen with a 25.4 mm diameter. Based on that result, the TCC can be obtained as 246.7 W/m^2^∙K. In reference to the studies, the TCC values between polymer and metal with various air pressures were between 40 and 270 W/m^2^∙K.

## 3. Thermal Behavior at Elevated Temperatures

### 3.1. Furnace Tests

**Figure 6:**
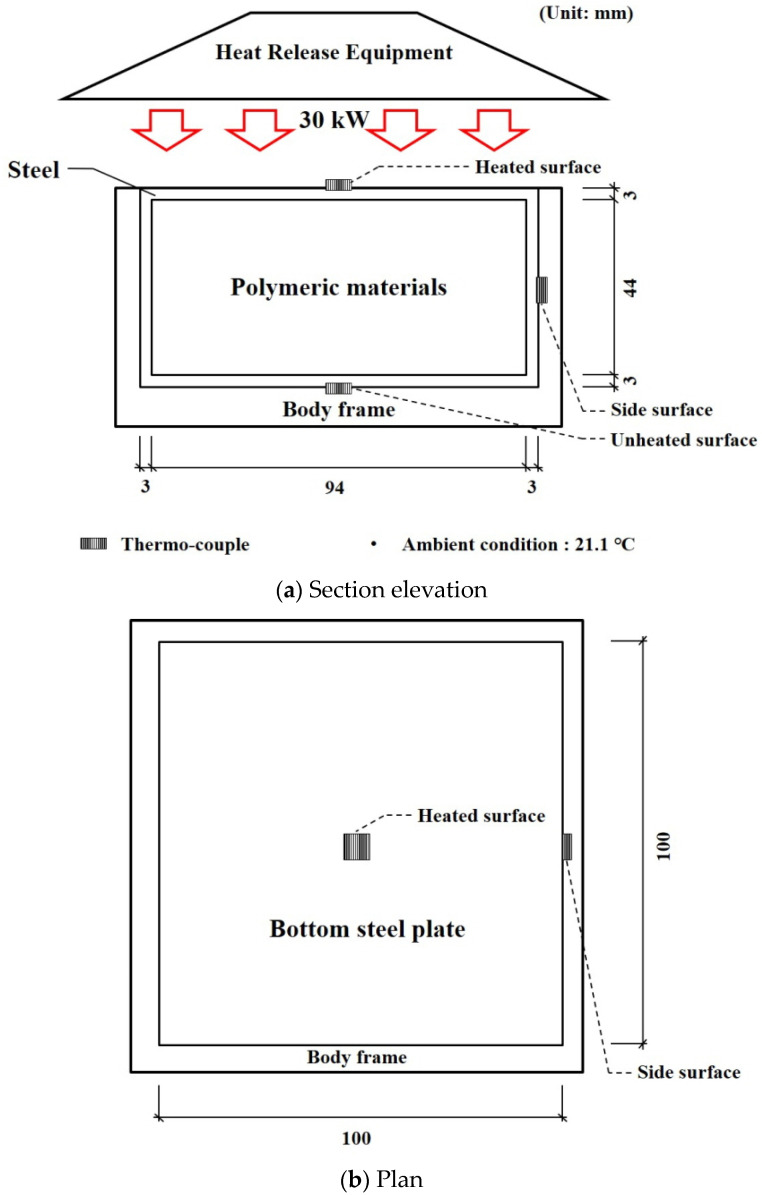
Set-up of furnace tests: (**a**) Section elevation (**b**) Plan.

**Figure 7:**
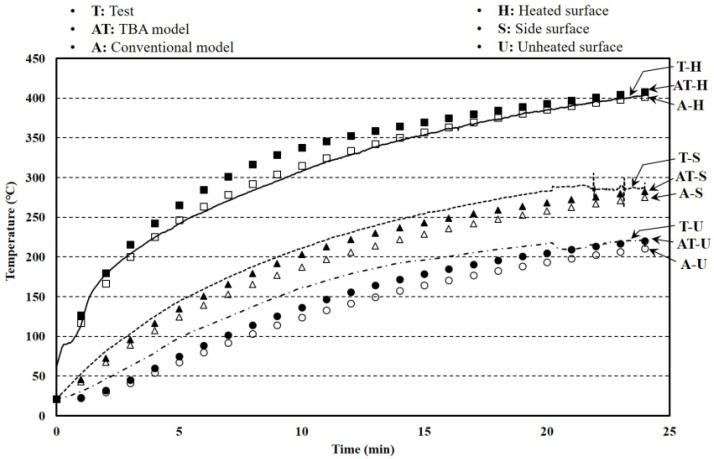
Temperature results of furnace tests and finite element analysis (FEA).

Furnace tests with a 0.01 m^2^ heated surface were conducted to investigate the fire resistance performance of the hybrid floor. The specimen size and setup of the furnace test known as a cone calorimeter [39,40,41,42,43,44,45,46] are shown in Figure 6. The aim of the tests was to effectively investigate the thermal behavior of the hybrid floor at elevated temperatures. The heated surface was uniformly heated at a rate of 30 kW for 24 min. The k-type thermo-couples that have 0.75% errors were installed at heated and unheated surfaces. The test was stopped when an unexpected excessive deformation occurred that was considered in the other tests. The temperature results of the heated, unheated, and side surface are shown in Figure 7.

### 3.2. Insulated Furnace Test

**Figure 8:**
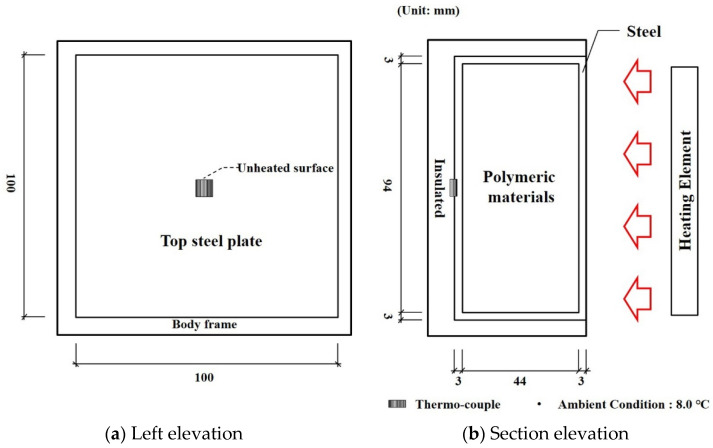
Setup of insulated furnace tests: (**a**) Left elevation (**b**) Section elevation.

**Figure 9:**
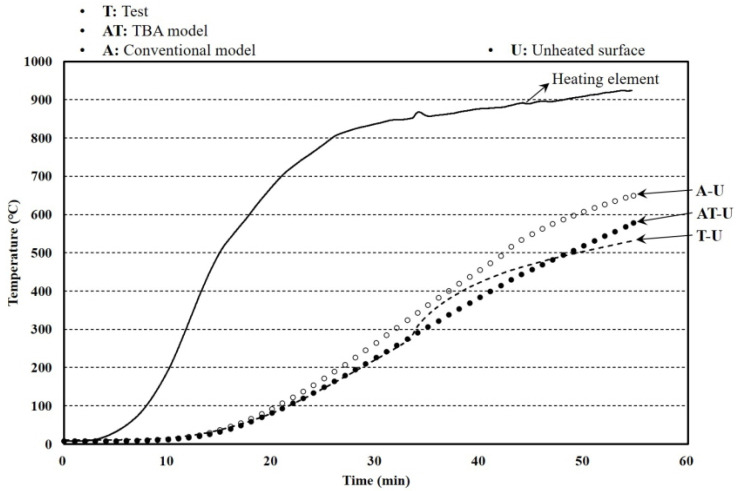
Temperature results of insulated furnace tests and FEA.

The difference from the previous furnace tests was the boundary conditions of the unheated and side surfaces. The unheated surfaces of this test were perfectly insulated, and this led to the specimen’s elevated temperature. The size of the specimen and the setup of the insulated furnace test are shown in Figure 8. The aim of the tests was to investigate the thermal behavior of the hybrid floor at a more extreme condition than that of the cone calorimeter tests because there was little significant temperature and phase change during the previous tests. The k-type thermo-couples that have 0.75% errors were installed at the unheated surface. The surface was heated for 54 min by the heating curve that targeted the standard curve, and the unheated and side surfaces were insulated as shown in Figure 9. Therefore, the temperature changes in the specimen showed large differences.

### 3.3. Thermal Behavior Based on the Furnace Tests

**Figure 10:**
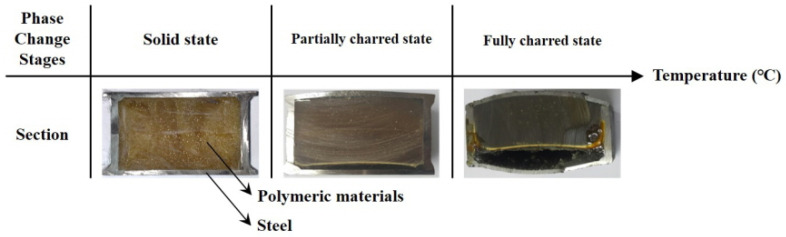
Three phase change stages at elevated temperatures.

The phase changes of the hybrid floor at elevated temperature were divided into three stages as shown in Figure 10. First, the polymeric materials in the floor were in the solid state. The first stage was called the solid state. In the solid state, the properties of the polymeric materials were controlled only by the temperatures. Second, the polymeric materials in the floor displayed rubber and liquid state, and the rubber state and liquefied polymeric materials turned into charred polymeric materials or air. The second stage was called the partially charred state. In the partially charred state, air created a large gap between the polymeric materials and heated surface. Finally, the charred polymeric materials created a layer between the solid-state polymeric materials and lower steel plates that were the heated surface in the tests. The third stage was called the fully charred state, and the thickness of the charred layer remained at 2–5 mm that was disclosed in the preliminary fire furnace tests. As this layer behaved as an insulation layer in fully charred state, the heat transfer from the heated surface to polymeric materials was weak, and the heat flow detoured to the sides or the weak spots in the charred layer. Based on the TGA results, the range of temperatures for charred were 370–500 °C, and the polymeric materials remained perfectly charred and in a gaseous state over 500 °C.

## 4. Verifications with Finite Element Analysis

**Figure 11:**
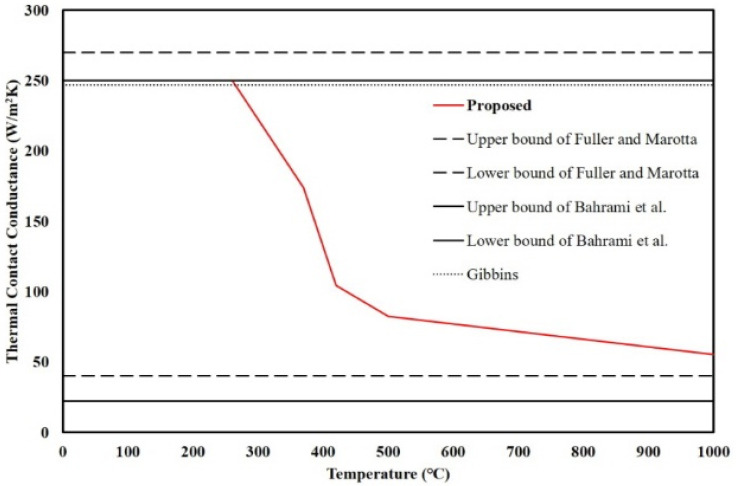
Thermal contact conductance between the steel and polymer at elevated temperatures.

**Figure 12:**
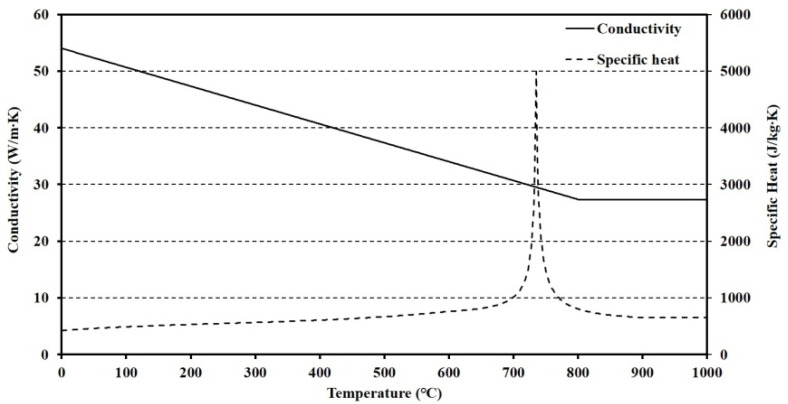
Conductivity and specific heat of steel at elevated temperatures.

**Figure 13:**
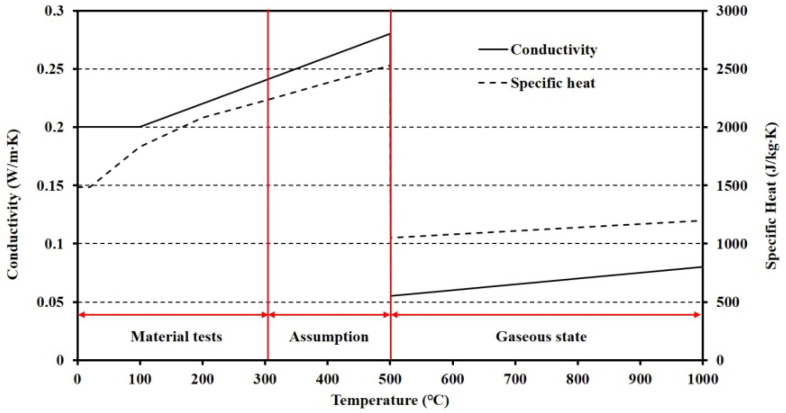
Conductivity and specific heat of polymeric materials at elevated temperatures.

To apply the thermal behavior to predict the temperature distribution of the floor, the reliability of the TBA model should be obtained. In this study, the reliability of the TBA model was obtained by an FEA that simulates furnace tests. The FEA program ABAQUS 2017 was used. The DC3D8, which is an 8-node linear heat transfer brick, was used to generate the meshes. The material properties including density, thermal conductivity, and specific heat at elevated temperatures of steel were obtained from Eurocode 3 [47]. Other material properties including density, thermal conductivity, and specific heat of polymeric materials below 300 °C were obtained from material tests and linear proportional assumptions based on the standards [48]. The tests, known as the flash method used to measure values of thermal diffusivity of a wide range of solid materials, based on the standards measure the thermal diffusivity that consists of specific heat, density, and thermal conductivity. The material properties of polymeric materials over 500 °C were assumed as air and obtained from a study [49]. The TCC between steel and polymer at ambient temperature was determined by previous studies [27,28,29] as 250 W/m^2^·K. The reduction ratio of TCCs at elevated temperatures followed the results of the TGA with insignificant modification based on FEA results and previous TCC studies between polymers and metal. The boundary conditions, including the heat transfer, convection, and radiation, at heated and unheated surfaces, were determined by several studies [47,50,51,52,53,54,55,56,57,58,59,60,61,62,63,64,65,66,67,68]. A summary of the material and interfacial properties is shown in Figure 11, Figure 12 and Figure 13. The proposed TCC at elevated temperatures in this study can be written as

(1) $$TCC(W/m^{2}\cdot K)=\{\begin{array}{cc} \begin{array}{l} 250\\ 250-0.6932(T-260)\\ 173.75-1.3875(T-370)\\ 104.38-0.2734(T-420)\\ 82.5-0.05438(T-500)\\ 55.31 \end{array} & \begin{array}{l} \left(T\leq 260℃\right)\\ \left(260℃<T\leq 370℃\right)\\ \left(370℃<T\leq 420℃\right)\\ \left(420℃<T\leq 500℃\right)\\ \left(500℃<T\leq 1000℃\right)\\ \left(1000℃<T\right) \end{array} \end{array}$$

where *T* is the temperature. The furnace tests were selected to verify the TBA model based on the FEA. In addition, other furnace tests had a 6.25 times larger heated surface and were used to obtain the reliability of the TBA model for larger-scale tests. The results of the conventional model that simulates the furnace tests without the TCC were also compared to determine the importance of the TCC in this study.

### 4.1. Furnace Tests

The boundary conditions showing the emissivity and convection coefficients in the FEA for the heated and unheated surfaces are listed in Table 2. The reason the convection at the unheated surfaces was not considered was because of the body frame that fixed the specimen surrounding the unheated surfaces as shown in Figure 6. The average errors of the analysis results from the TBA and conventional models compared with the test results were 9.60% and 15.90%, respectively, while the corresponding standard deviations of the errors were 14.09% and 13.44%, as shown in Table 3. The error reduction of the TBA model compared with the conventional model was 60.38%. The reason the errors showed these larger results was because the thermal lag time was ignored in the interstitial properties and this caused a significant difference at the early time [69]. The errors at a specific time can be written as

(2) $$\varepsilon_{time}(\%)=\left|\frac{\Delta T_{analysis}-\Delta T_{test}}{\Delta T_{test}}\right|=\left|\frac{\left(T_{analysis,time}-T_{initial}\right)-\left(T_{test,time}-T_{initial}\right)}{\left(T_{test,time}-T_{initial}\right)}\right|$$

where *T* is the temperature. In calculating the errors’ average and standard deviation, the unheated surfaces that significantly influenced the TBA model in this study were considered. The small temperature differences at the heated surfaces were generated by the initial temperature of the specimen because of the error in the thermocouples.

### 4.2. Insulated Furnace Tests

The boundary conditions showing the emissivity and convection coefficients in the FEA at the heated surfaces are listed in Table 2; moreover, the unheated surfaces were perfectly insulated. In the insulated furnace tests, only the temperatures of the furnace and unheated surface were measured due to a limitation of the furnace shown in Figure 8. The average error of the analysis results with the TBA and conventional models were 14.74% and 20.61%, respectively, while the error deviations were 24.30% and 23.26%, as listed in Table 3. The error reduction of the TBA model compared with the conventional model was 71.52%. The average error reduction was 65.95%. Based on the verification results of the two furnace tests, the TBA model proved to be a valid thermal model to predict the temperatures of the hybrid floor at elevated temperatures.

### 4.3. Larger Furnace Tests

**Figure 14:**
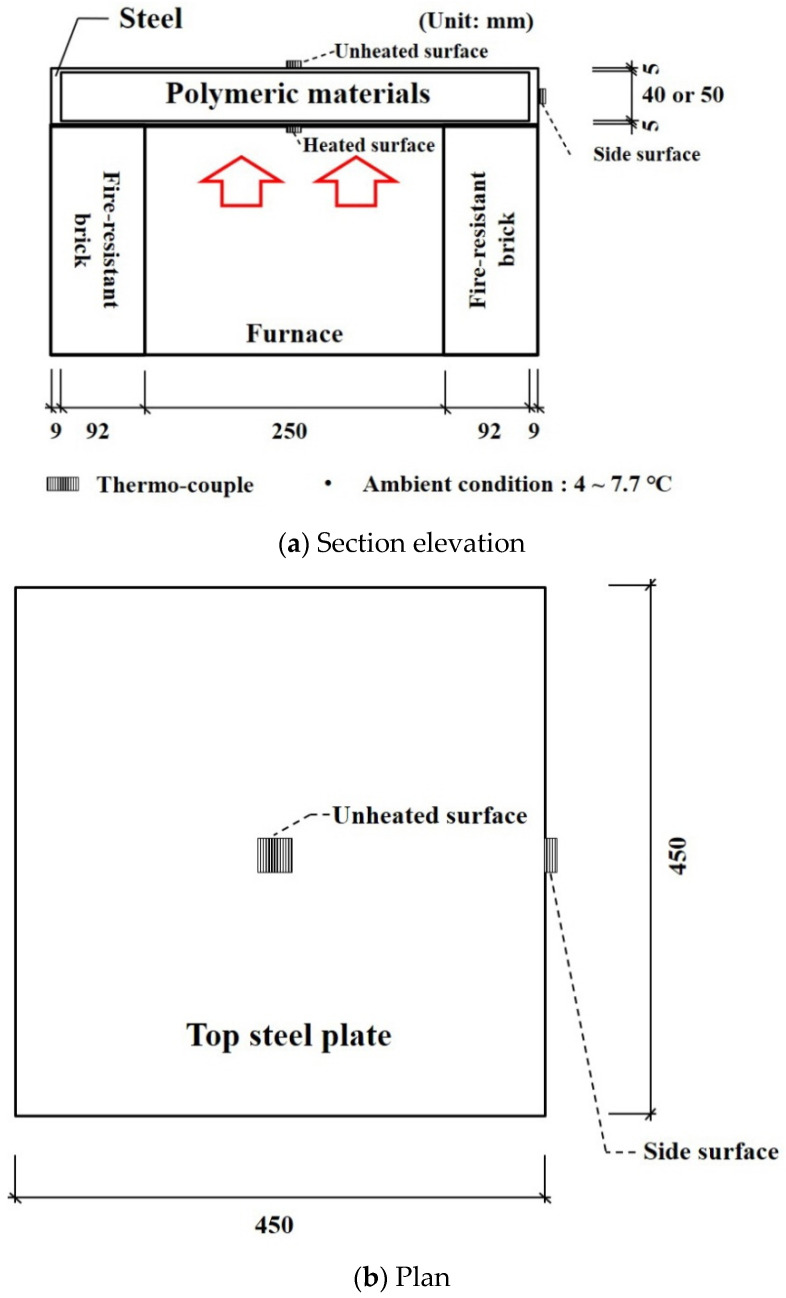
Setup of larger furnace tests: (**a**) Section elevation (**b**) Plan.

**Figure 15:**
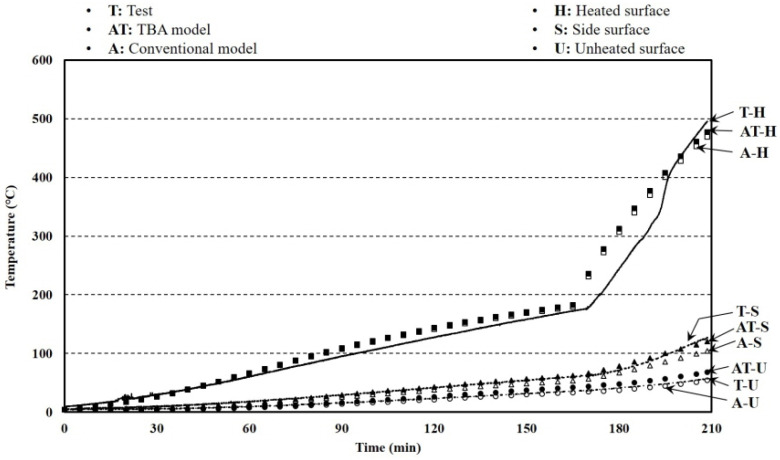
Temperature results of larger furnace tests and FEA with a 50-mm thickness.

**Figure 16:**
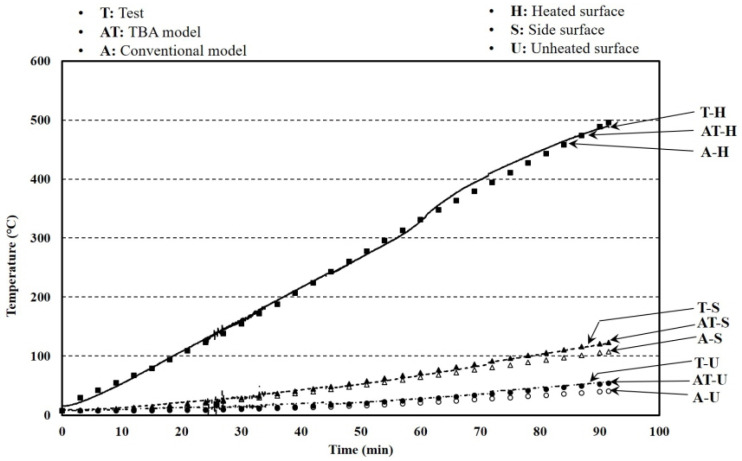
Temperature results of larger furnace tests and FEA with a 60-mm thickness.

The aim for verifying the larger furnace test results was to obtain the reliability of predicting the temperature distribution of a larger-sized floor with the FEA results. The specimen size and the test setup are shown in Figure 14. The k-type thermo-couples that have 0.75% errors were installed at heated, unheated, and side surfaces. The boundary conditions showing the emissivity and convection coefficients in the FEA at the heated and unheated surfaces are listed in Table 2. As the heating capacity of the larger furnace was irregular, two tests results were used. The test with a 60-mm specimen thickness showed the largest heat capacity, and the other test that had a 50-mm specimen thickness took the longest time to complete. The test results and analysis are shown in Figure 15 and Figure 16. The average errors for the 50-mm analysis results with the TBA and conventional models were 20.99% and 23.77%, respectively, while the corresponding error deviations were 23.49% and 24.63%. The average errors for the 60-mm analysis results with the TBA and conventional models were 26.03% and 35.86%, respectively, while the corresponding error deviations were 33.31% and 32.58%. The error reductions for the 50- and 60-mm TBA models compared with the conventional model were 88.30% and 72.59%, respectively. The entire average error reduction including the smaller furnace tests was 73.20%. The reliability of the TBA model applied to larger furnaces that was proposed for smaller furnace tests was obtained by the above results.

## 5. Conclusions

A hybrid floor was developed as a prefabricated floor system for steel structures. Based on previous studies, the floor has a good structural performance and serviceability. To be a more effective floor that can be applied to actual buildings without insulating materials for fire prevention, the floor should show an acceptable fire resistance performance as evaluated through tests conducted in Korea based on the KS. As the fire tests demand enormous costs and time to conduct, the number of specimen variables was limited. Therefore, more efficient methods such as the FEA and furnace tests that allowed for numerous variables were performed in this study to investigate the thermal behavior of the hybrid floor at elevated temperatures. In addition, FEAs with ABAQUS 2017 were performed to verify the TBA model, and larger furnace test results were used to verify the effect on larger-sized floors. In accordance with the furnace tests and FEA, the following points summarizes the results presented herein.

(1) The application of TCC related to the contact issues between two materials for predicting the time history of temperature distributions obtained more reliable analysis results compared with the results without considering TCC. The analysis results with TCC showed errors reduced by 73.20% compared with the results without TCC.
(2) When the TCC between polymeric materials and steel at elevated temperatures could not be measured, it can be determined by the results of the TGA that was reliable deduction based on previous studies and the analysis results.
(3) The proposed TBA model showed average errors of 17.84% in the analysis results compared with the time history of tests results. This means the proposed analytical modelling was a reliable way to predict the temperature distribution of the hybrid floor at elevated temperatures.
(4) When the polymeric materials exhibited thermal properties and TGA results analogous with the ones in this study, the TCC at elevated temperatures with steel in Equation (1) could be used to calculate the temperature distribution in the elements that contained contact problems between polymeric materials and steel.

## Figures and Tables

**Table 1:** Mechanical properties of the polymeric materials [3].

Property	Value
ρ: Density (kg/m^3^)	1178
σ_t_: Tensile strength (MPa)	31.4
E_t_: Modulus of elasticity in tension (MPa)	1277
σ_c_: Compressive strength (MPa)	23.1
E_c_: Modulus of elasticity in compression (MPa)	461
ν: Poisson’s ratio	0.39

**Table 2:** Boundary conditions of furnace tests in FEA.

Tests	Heated Surface			Unheated Surface		
	Convective Coefficient (W/m^2^∙K)	Emissivity of Fire	Surface Emissivity of Member	Convective Coefficient (W/m^2^∙K)	Emissivity of Fire	Surface Emissivity of Member
Furnace tests	10	1	0.7	-	1	0.7
Insulated furnace tests	25	0.8	0.7	Insulated		
Larger furnace tests (Thickness: 50 mm)	25	1	0.7	4	1	0.7
Larger furnace tests (Thickness: 60 mm)	120	1	0.9	4	1	0.7

**Table 3:** Errors average and deviation of analysis results compared with the test results.

Tests	TBA Model		Conventional Model		Error Reduction (m_wT_) /(m_woT_)
	Average (m_wT_)	Standard Deviation	Average (m_woT_)	Standard Deviation	
Furnace tests	9.60%	14.09%	15.90%	13.44%	60.38%
Insulated furnace tests	14.74%	24.30%	20.61%	23.26%	71.52%
Larger furnace tests (Thickness: 50 mm)	20.99%	23.49%	23.77%	24.63%	88.30%
Larger furnace tests (Thickness: 60 mm)	26.03	33.31%	35.86%	32.58%	72.59%
